# A Holistic Approach to Expressing the Burden of Caregivers for Stroke Survivors: A Systematic Review

**DOI:** 10.3390/healthcare12050565

**Published:** 2024-02-29

**Authors:** Eftychia Tziaka, Anna Tsiakiri, Pinelopi Vlotinou, Foteini Christidi, Dimitrios Tsiptsios, Nikolaos Aggelousis, Konstantinos Vadikolias, Aspasia Serdari

**Affiliations:** 1 Neurology Department, Democritus University of Thrace, 68100 Alexandroupolis, Greece; etziaka@med.duth.gr (E.T.); fchristidi@med.uoa.gr (F.C.); kvadikol@med.duth.gr (K.V.); 2 Department of Occupational Therapy, University of West Attica, 12243 Athens, Greece; pvlotinou@uniwa.gr; 3 3rd Department of Neurology, Aristotle University of Thessaloniki, 54124 Thessaloniki, Greece; dtsiptsi@med.duth.gr; 4 Department of Physical Education and Sport Science, Democritus University of Thrace, 69100 Komotini, Greece; nagelous@phyed.duth.gr; 5 Department of Child & Adolescent Psychiatry, Medical School, Democritus University of Thrace, 68100 Alexandroupolis, Greece; aserntar@med.duth.gr

**Keywords:** burden, stroke, rehabilitation, care

## Abstract

This systematic review explores the multifaceted challenges faced by caregivers of stroke survivors, addressing the global impact of strokes and the anticipated rise in survivors over the coming decades. Following the Preferred Reporting Items for Systematic Reviews and Meta-Analyses (PRISMA) guidelines, a thorough literature search identified 34 relevant studies published between 2018 and 2023. The review categorizes caregiver burden into four domains: physical health, social functioning, financial issues, and psychological health. Caregivers often experience a decline in physical health, marked by chronic fatigue, sleep disturbances, and pain. Emotional distress is prevalent, leading to anxiety and depression, especially in cases of high burden. Financial strains arise from medical expenses and employment changes, exacerbating the overall burden. Contextual factors, such as cultural norms and resource availability, influence the caregiver experience. The Newcastle–Ottawa scale assessed the methodological quality of studies. The conclusion emphasizes tailored interventions and support systems for caregivers, with practical recommendations for healthcare professionals, therapists, mental health professionals, financial counselors, and policymakers. This comprehensive review enhances the understanding of caregiver experiences and provides actionable insights to improve stroke care and rehabilitation The study’s novelty lies in its holistic examination of caregiver burden in stroke care, its focus on the recent literature, and its emphasis on forecasting caregiver outcomes, contributing valuable insights for proactive intervention strategies.

## 1. Introduction

Strokes are a considerable challenge for healthcare worldwide. Despite significant advances in early diagnosis and treatment, stroke remains the second leading cause of death globally, after heart disease, as well as the primary cause of disability among adults [1,2]. Moreover, in light of stroke’s age-related nature [3], with more than half of stroke patients being over 65 [4], and the ever-expanding lifespans of individuals, which will nearly triple the number of cases in adults over 60 by 2050 [5], stroke survivors are expected to increase dramatically over the following decades. Furthermore, stroke events are considered a continuum, as their effects are similar to chronic diseases because most deficiencies cannot be restored to pre-stroke levels [6]. As a result, the majority of stroke survivors who return to the community after a stroke will exhibit some disabilities or experience some difficulties [7], and they will have to rely on others to care for them.

After the patient’s hospitalization, in settings where formal post-discharge management and support are not provided, family members and relatives take over the informal role of caring for the stroke survivor at home [8]. Considering these parameters, life after a stroke is often quite challenging for informal caregivers as the sudden onset of disabilities and the chronic, often unforeseen nature of stroke recovery place a significant burden on their shoulders [9,10]. Caring for a patient at home has been considered a stressful event impacting the caregivers’ physical and mental health, quality of life, economic stability, and social relations and interactions [11].

To be more thorough, when informal caregivers take on this challenging and unprecedented role, they bear several responsibilities fraught with burdens. Specifically, several studies have highlighted that caregivers often exhibit signs of tension, anxiety, depression, and stress [12,13,14]. Based on a meta-analysis of 34 studies, about 40% of caregivers who assist and provide care to stroke survivors experience depressive symptoms at various points during the assessment process [15]. Furthermore, researchers have examined the progress of depression symptoms during the early stages of caregiving, and 20% to 40% of caregivers report such symptomatology during the first three months of caring for the patient [16,17,18]. In long-term care for stroke survivors, it was noted that caregivers’ mild depression symptoms might become more severe or persistent if they do not manage them.

Alongside the mental health problems that carers may experience, physical problems can manifest themselves. Such difficulties make it more difficult for them to help the patient and reduce their quality-of-life levels. On the one hand, caregivers feel weak and, over time, tend to give up their efforts to care for the patient as they seem to believe that they cannot cope any longer with the challenges brought about by the situation. On the other hand, however, they feel a deep sense of obligation towards the patient, especially if they are relatives within the close family circle. In this case, despite all the burdens, they continue to provide their informal services, compromising their own physical and mental health [19].

In continuation, apart from the psychological strains, a caregiver could be burdened by economic-related hurdles. For example, according to a study based in the UK, when informal caregiving costs were included, the cost of covering all expenses escalated to GBP 9405 from GBP 15,306 over five years [20]. These exorbitant amounts of money do not, in most cases, correspond to the financial capacity of the caregivers who, in addition to their own needs, have to cover the expenses related to the care and rehabilitation of the patient. Finally, it is essential to highlight that the lack of social support and personal interactions with individuals outside the patient’s setting could potentially increase the already existing burden of the carer, making them the “second patient” in the house [21].

The scientific explanation detailing the factors behind the correlation between caregiver burden and its influence on stroke patients is intricate and fundamental for understanding the imperative nature of investigating this phenomenon. Firstly, the recovery and well-being of stroke patients are heavily dependent on the quality of care they receive, particularly in the post-hospitalization period where informal caregivers assume primary responsibility. Research consistently demonstrates that higher levels of caregiver burden are associated with poorer patient outcomes, including increased risk of depression, slower recovery, and reduced quality of life for stroke survivors [22,23].

Moreover, the stress and strain experienced by caregivers can directly translate into suboptimal care for the stroke patient. Caregiver distress may lead to neglect of the patient’s needs, medication errors, and inadequate support for rehabilitation activities, all of which can impede the patient’s recovery trajectory [24,25]. Additionally, caregivers experiencing significant burdens may struggle to maintain the necessary patience, empathy, and encouragement needed to support the stroke survivor effectively through their rehabilitation journey.

Furthermore, the reciprocal nature of the caregiver–patient relationship intensifies the significance of caregiver burden. As caregivers experience heightened levels of stress, depression, and physical strain, their ability to provide consistent and high-quality care diminishes, thereby exacerbating the challenges faced by the stroke patient. This cycle of reciprocal influence underscores the critical importance of addressing caregiver burden to optimize outcomes for both the caregiver and the stroke survivor [26,27].

The present study aims to address the global issue of caregiver burden in the context of stroke survivors. Given the compelling evidence linking stroke patients and the burden experienced by their caregivers, there is an emerging need to accurately forecast caregivers’ susceptibility to depression, diminished quality of life, social distancing, and isolation. In line with this, the study endeavors to review all the pertinent literature published within the last five years that explores the potential outcomes associated with caregivers’ burden when dealing with stroke survivors. By documenting the known effects of this situation, the study seeks to shed light on the multifaceted challenges faced by caregivers worldwide, emphasizing the global significance of the caregiver role in stroke care. This study’s novelty lies in its holistic examination of the global issue of caregiver burden in stroke care. It integrates diverse perspectives from recent literature, capturing emerging trends and identifying gaps in understanding. Unlike prior research, this study takes a holistic view of caregiver well-being, considering psychological, physical, economic, and social factors. It uniquely emphasizes forecasting caregiver outcomes, enabling proactive interventions to address distress and enhance overall well-being.

The choice of the years 2018 to 2023 for the systematic review was based on several scientific considerations. Firstly, this timeframe ensures that the review captures the recent literature, providing up-to-date insights into the challenges faced by caregivers of stroke survivors. Given the dynamic nature of healthcare research, focusing on recent publications allows for the inclusion of emerging trends and findings that may not be reflected in older studies. Additionally, restricting the search to a specific period helps maintain the relevance and applicability of the review’s conclusions to current clinical practice and policy development. Moreover, selecting a relatively narrow timeframe facilitates a manageable and comprehensive review process, ensuring thorough coverage of the available literature within a reasonable scope. By concentrating on a five-year period, the review can strike a balance between inclusivity and feasibility, optimizing the efficiency of data collection and analysis.

## 2. Materials and Methods

The current review was conducted and reported with the guidance of the Preferred Reporting Items for Systematic Reviews and Meta-Analyses (PRISMA) statement [22] (PROSPERO registration number: CRD42024504258).

### 2.1. Search Strategy

Three investigators (ET, AT, and PV) conducted a literature search of two databases (PubMed and Scopus) to trace all relevant studies published between 1 January 2018 and 20 January 2023. Search terms were as follows: (“stroke” AND “caregivers” AND “burden”). Table 1 lists the structured search techniques for each database. The retrieved articles were also manually searched for any further potentially eligible articles. Any disagreement regarding the screening or the selection process was discussed with a fourth investigator (AS) until a consensus was reached.

### 2.2. Search Criteria

Only original full-text articles published in the English language were included. Furthermore, the sample of articles included in the systematic review consisted of individuals aged 19 and older. Secondary analyses, reviews, guidelines, meeting summaries, comments, unpublished abstracts, studies conducted with animals, and studies focused on the patients’ perspectives of stroke burden were excluded. There were no restrictions on the study design or sample characteristics.

### 2.3. Data Extraction

Data extraction was performed using a predefined data form created in Excel. We recorded the authors, the year of publication, the title of each article, the type of study, the number of participants, demographic data (age, gender, hours the caregiver spent with the stroke survivor, if the caregiver was living with the stroke survivor), how much time had passed after the stroke, the type of caregiver, the type of relationship between the caregiver and the stroke survivor, the psychometric scales used to measure caregiver burden, possible limitations of the studies, and the main findings.

### 2.4. Data Quality Assessment

We accessed the risk of bias using the Newcastle–Ottawa scale (NOS) for included non-randomized studies (https://www.ohri.ca, assessed on 7 May 2023). NOS uses a ‘star system’ in which a study is judged on three elements: the selection of the study groups, the comparability of the groups, and the ascertainment of either the exposure or outcome of interest. A study can be awarded a maximum of one star for each item within the Selection and Exposure categories and a maximum of two stars for the Comparability section. A total quality score (maximum of 9 stars) was calculated based on the sum of stars allocated. All quality assessments were conducted independently in duplicate (A.T., P.V.) and reviewed with a senior team member (K.V.). The sum of points for all items was used to categorize overall study quality as either high (>7), moderate (5–7) or low (<5) to determine the reliability of the outcome reports [23]. No statistical analysis or meta-analysis was performed due to the high level of heterogeneity among the studies. Thus, the data were only descriptively analyzed.

## 3. Results

**Figure 1:**
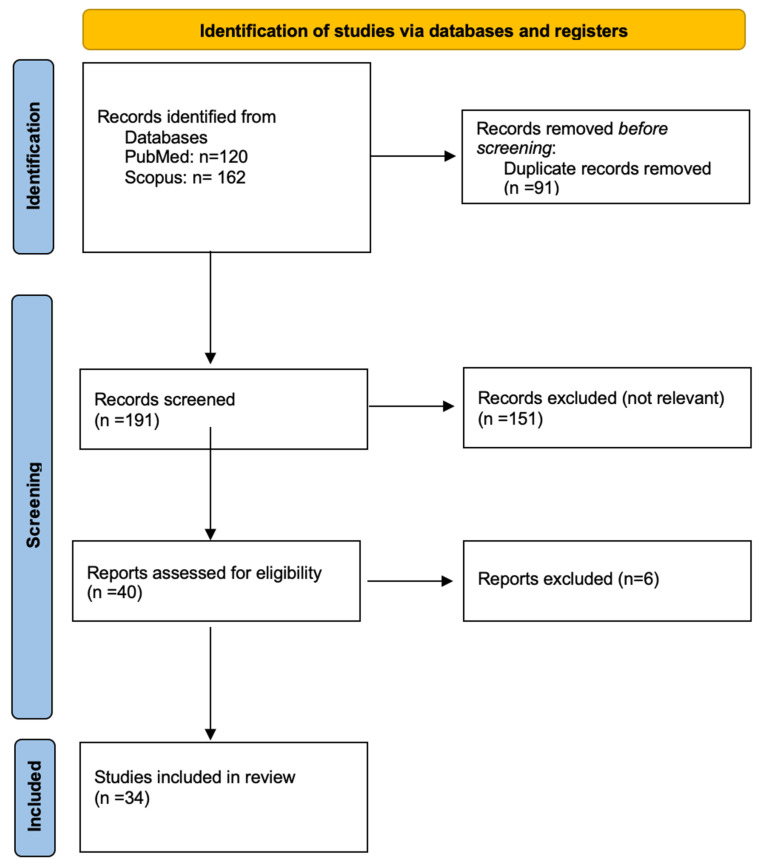
Prisma flow-chart.

The process of study and the selection of included studies are summarized in Figure 1. A total of 282 articles were identified from the two databases. After removing the duplicates (*n* = 91), 191 articles were retained to check their relevance with the aim of the present study. Titles and abstracts were checked for relevance, and 151 articles were excluded. According to the selection criteria, 6 more articles were excluded, while 34 met eligibility and were included in the review.

### 3.1. Risk of Bias Assessment

Quality assessment is summarized in Table 2. The median score was 5–7 stars (*n* = 21), while the highest was over seven total stars (*n* = 9) and the lowest was under 5 total stars (*n* = 4). The high risk of bias was mainly found in the following individual items: “Selection—non-exposed cohort” (5/34 stars), “Outcome—length of follow-up” (9/34 stars) and “Outcome—adequacy of follow-up” (9/34 stars).

### 3.2. Study Characteristics

**Figure 2:**
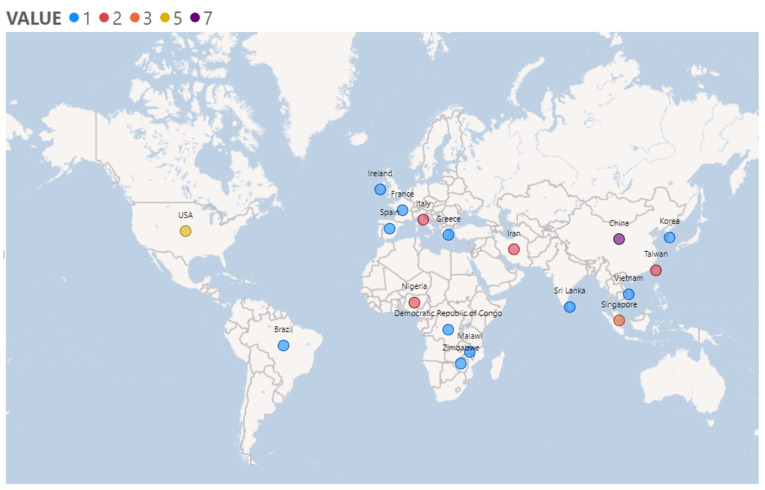
Studies origins. Value: number of studies per country.

A summary of the included studies is presented in Table 3. All studies were published between 2018 and 2022 and conducted in 18 different countries, namely, China (*n* = 7), Nigeria (*n* = 2), Vietnam (*n* = 1), Italy (*n* = 2), Sri Lanka (*n* = 1), Taiwan (*n* = 2), Brazil (*n* = 1), USA (*n* = 5), Greece (*n* = 1), Iran (*n* = 2), France (*n* = 1), Malawi (*n* = 1), Zimbabwe (*n* = 1), Singapore (*n* = 3), Democratic Republic of Congo (*n* = 1), Korea (*n* = 1), Ireland (*n* = 1) and Spain (*n* = 1) (Figure 2).

### 3.3. Characteristics of Participants

A total of 4068 stroke caregivers were included in this review, with mean ages ranging from 28.2 years to 64.9 years. The sample size ranged from 8 to 361 and only consisted of informal caregivers. The relationship status was not specified in all studies but mainly were family members and characterized as the primary caregiver of stroke survivors. The timing of the study in relation to the stroke event varies in the included studies over a range that includes the acute phase (day 1) up to the chronic phase (102 months). Regarding the main characteristics of caregivers, a variety of differences between surveys have been recorded. Those identified in the majority of studies are related to the gender of caregivers, with a higher proportion of female caregivers (range: 62.2–70.3%), most caregivers living with the stroke survivor (up to 83.3%), the average daily time needed to care for the stroke survivor (range < 4 h–>13 h), the average time the caregiver has been in the role (range: 3 weeks–>2.5 years).

### 3.4. Measurement Scales for Burden

Structured rating scales (*n* = 26) and semi-structured interviews (*n* = 8) were used to measure burnout. The scales used in most studies are the Zarit Burden Interview Scale (*n* = 13), other scales measuring burden (*n* = 3), the Caregiver Strain Index (*n* = 3), and the Multidimensional Scale of Perceived Social Support (*n* = 3).

### 3.5. Domains of Burden Expression

After conducting a systematic review of relevant literature from 2018 to 2023, this study provides comprehensive insights into the multifaceted challenges faced by caregivers of stroke survivors. The selection of these four domains—emotional, physical, social, and financial—reflects a holistic approach to understanding caregiver burden in the context of stroke care. Each domain represents a distinct aspect of caregivers’ experiences and challenges, encompassing a wide range of factors that can impact caregivers’ well-being and ability to provide effective care for stroke survivors

In the emotional domain, caregivers commonly experience heightened levels of tension, anxiety, depression, and stress. The sudden onset of disabilities and the chronic, often unforeseen nature of stroke recovery place a significant burden on caregivers’ mental health. Research indicates that approximately 40% of caregivers assisting stroke survivors experience depressive symptoms, with symptoms persisting or worsening over time if not adequately managed.

In the physical domain, caregivers frequently report a decline in physical health, marked by chronic fatigue, sleep disturbances, and pain. These physical symptoms can significantly impair caregivers’ ability to provide optimal care and support for stroke survivors, further exacerbating their overall burden.

In the social domain, caregivers often face challenges related to social functioning and support. The lack of social support and personal interactions outside the patient’s setting can exacerbate caregivers’ feelings of isolation and contribute to their sense of being the “second patient” in the household. Moreover, cultural norms and resource availability can significantly influence the caregiver experience, adding additional layers of complexity to their social interactions.

In the financial domain, caregivers may encounter economic-related hurdles, including medical expenses and changes in employment status. The financial strain of caregiving can exacerbate the overall burden, particularly if caregivers are unable to cover the costs associated with care and rehabilitation. Research indicates that informal caregiving costs can escalate significantly over time, placing additional financial stress on caregivers and compromising their financial stability.

Overall, the study’s findings underscore the holistic nature of caregiver burden in the context of stroke care, emphasizing the interconnectedness of emotional, physical, social, and financial challenges faced by caregivers worldwide. These insights highlight the urgent need for tailored interventions and support systems to address the diverse needs of caregivers and improve stroke care and rehabilitation outcomes (see Table 4).

## 4. Discussion

### 4.1. The Impact of Caregiver Burden on Physical Health among Stroke Caregivers

Caring for stroke survivors entails a complex web of responsibilities that often take a toll on caregivers’ physical health. Long [25], Tsai [30], and Marima [46] converged on the idea that caregiver burden is a significant predictor of a decline in overall health. The burden of caregiving appears to catalyze physical health issues, ranging from chronic fatigue to sleep disturbances. This common thread underscores the pervasive impact that caregiver burden can exert on caregivers’ physical well-being. More specifically, Long [25] found that caregivers of stroke survivors often experience a decline in their overall health, with caregiver burden being a strong predictor of this decline. The study emphasized that caregivers facing higher levels of burden tend to struggle with coping mechanisms, increased worry about the care recipient, and reduced social involvement. The degree of dependency of the stroke survivor on the caregiver’s assistance for daily living tasks also contributes to the caregiver’s physical and mental well-being. Tsai [30] highlighted the strong connection between caregivers’ self-rated health status and their overall quality of life (QoL). Caregivers’ own health status played a mediating role in the relationship between burden and QoL. Physical and mental strains experienced by caregivers, including back pain, poor sleep quality, and fatigue, significantly affected their QoL. Marima [46] discussed the prevalence of physical health problems, such as chronic fatigue, pain, and poor lifting techniques, among caregivers in low-resourced settings. These physical challenges were linked to poor mental health outcomes.

Several studies, including Caro [19] and Farahani [40], collectively paint a picture of caregivers grappling with physical challenges such as exhaustion, pain, and sleep alterations. The physical demands of assisting stroke survivors in their activities of daily living seem to exacerbate caregivers’ fatigue and discomfort. This shared narrative underscores the tangible physical strains that caregivers encounter. The study of Caro pointed out the prevalence of physical issues among caregivers, including chronic fatigue, pain, and psychosomatic problems. The physical demands of caregiving, such as assisting with activities of daily living, were noted as potential contributors to these health problems. Outcomes of Farahani’s research emphasized that caregivers also face physical problems like fatigue, sleep disturbances, exhaustion, and pain, alongside emotional challenges such as worry and anxiety.

The interplay between caregivers’ self-rated health and their burden is another recurrent theme [30,35,37] converging on the notion that caregivers with better self-rated health tend to experience a lower burden. Conversely, poorer self-rated health intensifies the burden experienced by caregivers, leading to a cyclic relationship. This interdependence between health status and burden is a consistent finding across the studies. Lee [39] introduced a contextual difference by discussing the impact of the COVID-19 pandemic. The closure of support services and increased caregiving demands during the pandemic contributed to heightened physical burden and fatigue among caregivers. This unique context illuminated how external factors, such as a global crisis, can magnify the physical strain on caregivers.

While most studies focused primarily on the direct impact of burden, Formica [43] discussed the role of psychosocial factors in the caregiver’s physical well-being. The study explored how caregiver burden and distress influenced cognitive performance, indirectly affecting physical health. This distinction underscores the multifaceted nature of caregiver well-being, where psychosocial factors interact with the physical domain. The studies, including Hu [27] and Marima [46], highlight potential cultural and resource-related differences. Caregivers in low-resourced settings reported challenges due to a lack of appropriate aid and ergonomic training. This highlights how caregiving experiences and physical outcomes can differ based on cultural norms and available resources.

The collective findings from these studies underline the convergence of caregiver burden as a pivotal factor impacting the physical health of those caring for stroke survivors. However, the studies also reveal differences shaped by contextual factors, psychosocial intricacies, and resource availability. This nuanced understanding enhances our ability to develop tailored interventions and support systems that address both common challenges and unique circumstances faced by stroke caregivers.

### 4.2. The Impact of Burden on Social Functioning in Stroke Caregivers

The studies discussed [19,27,28,29,31,37,40,53] the impact of burden on social functioning in stroke caregivers and several common themes emerge. A prevailing similarity is the negative emotional toll on caregivers. Prolonged care durations often lead to heightened levels of anxiety and depression, attributing these feelings to the significant time caregiving demands, which subsequently reduces the caregivers’ personal, work, and social engagements. This emotional strain is compounded by the burden caregivers experience, regardless of whether it is tied to personal factors or responsibilities. This shared burden manifests in caregivers’ susceptibility to anxiety and depression. Moreover, the studies consistently highlight that caregivers’ lack of professional knowledge regarding stroke exacerbates this burden, leading to an increased investment of time and energy in the caregiving process and consequent mental health issues. Overall, the collective findings underline the emotional distress caregivers face and its direct association with the time-intensive nature of caregiving and the related burdens.

While the studies converge on several common aspects, there are notable differences that offer valuable insights into the diverse experiences of stroke caregivers. One such distinction is the cultural perspective. Wagachchige Muthucumarana’s study [29] delves into the Sri Lankan context, revealing that caregiving responsibilities curtail caregivers’ participation in cultural, social, and religious activities that hold normative significance in that society. Another contrast arises from the sources of social support. Akosile’s [31] work underscores the differential expectations from family and friends. While friends’ and significant others’ support is often appreciated for its flexibility, family members are sometimes expected to share the caregiving burden, leading to varying support dynamics. Additionally, the impact on sleep and leisure time, as highlighted in Caro and Mei, reveals a unique difference. This reduction stems from the challenges of balancing caregiving responsibilities, daily activities, and the absence of familial support. Notably, Mei [53] and Farahani [40] elucidate potential positive outcomes, showcasing that caregiving might foster improved interpersonal relationships and the opportunity to uphold traditional family values. Collectively, these differences emphasize the influence of culture, support networks, and contextual factors on the multifaceted experiences of stroke caregivers.

### 4.3. The Impact of Burden on Financial Issues in Stroke Caregivers

Several studies have investigated the impact of financial burdens on caregivers of stroke patients. The economic strain caused by stroke-related expenses and changes in employment status has significant implications for caregivers’ well-being. The economic burden on stroke caregivers is influenced by the family’s original economic situation and the medical payment method. Caregivers from families with medical insurance payments experience less anxiety and depression compared to self-paid families. Economic burden emerged as a significant factor influencing caregiver anxiety and depression. Implementing comprehensive caregiver support programs is recommended [27].

Financial difficulties resulting from reduced family income and high expenses lead to discontinuation of treatment and rehabilitation for stroke patients. Lack of awareness and information about government support exacerbates the issue. Financial constraints and insufficient knowledge about available support services are identified as major burdens for family caregivers [29]. Caregiver quality of life (QoL) is negatively affected by lower family income, especially among those caring for first-time stroke patients. Financial strain and quitting jobs to fulfil caregiver roles contribute to the challenges faced by home caregivers. Spouse caregivers, often ageing individuals, experience higher financial strain and negative well-being impacts compared to non-spouse caregivers [30].

Caregivers’ employment is significantly impacted by stroke care responsibilities. A considerable portion of caregivers leave employment or reduce work hours to provide care. The combination of reduced family income and increased healthcare expenses worsens the financial burden, compromising caregivers’ quality of life [19]. Caregivers with better financial resources tend to experience a lighter burden. The type of health insurance does not substantially reduce caregiver burden due to limited reimbursement content. Low insurance reimbursement rates contribute to the financial strain experienced by caregivers [35]. Prolonged hospitalization leads to increased financial burden on caregivers due to expenses associated with hospital visits and increased susceptibility to infections. These factors contribute to heightened caregiver needs during extended hospital stays [40]. The first year after a stroke is associated with substantial productivity losses and increased indirect costs. The level of patient dependence is positively correlated with higher productivity losses and indirect costs [42]. Caregivers’ ability to return to work is crucial for family financial stability. Interference between caregiving responsibilities and employment negatively affects family life and creates financial strain [45]. A significant portion of caregivers face inadequate finances, with lower income being linked to a higher risk of psychiatric morbidity. The added financial burden exacerbates the economic challenges of caregiving, contributing to a cycle of poverty that further elevates the risk of psychiatric morbidity [46].

These studies underscore the substantial impact of financial burdens on stroke caregivers. The economic challenges arising from medical expenses, reduced employment opportunities, and lack of financial support contribute to increased caregiver stress, reduced quality of life, and heightened risk of psychiatric morbidity. Addressing these financial burdens through improved support systems and comprehensive caregiver programs is crucial for enhancing both caregiver well-being and patient outcomes.

### 4.4. The Impact of Caregiver Burden on the Psychological Health among Stroke Caregivers

Several articles in the published literature focus on the burden on caregivers of stroke patients. The effects of this strain accompanying the patient’s care manifest in various forms. Regarding the psychological aspect, many articles report a significant positive correlation between the caregiver’s burden and the development/manifestation of depressive and anxiety symptoms.

Researchers report that caregivers with moderate to high levels of subjective burden experience or are at high risk for developing depressive symptoms [34,41,51] and exhibit emotional changes [19]. In addition, a cohort study by Koh and his colleagues [48] showed that caregivers who mentioned that were burdened by their caregiving responsibilities had high depression scores when their depressive symptoms were measured three months after the patient’s stroke. These scores appeared to decrease statistically significantly when assessed one year after stroke. Furthermore, another research study highlighted the fact that caregivers who had depressive symptoms and high levels of subjective burden in the early stages of their caregiving duties were at a high risk of having persistent depressive symptomatology throughout the first year of caregiving [51]. One in three caregivers tend to have high depressive symptoms during the first year of caring for stroke survivors. On the contrary, if the caregivers did not have a history of depression or exhibiting this type of symptoms whilst taking care of the patient, the possibility of them developing depression and high burden levels was low. Moreover, Caro et al. [19] noted that 86.7% of the caregivers who participated in her study exhibited symptoms of psychiatric problems and had a substantial burden score.

In continuation, apart from the risk of depression, there is the possibility of exhibiting anxiety symptoms when there are high levels of perceived burden [27,37]. Specifically, Kitoko et al. [49] noticed that high burden levels were associated with high anxiety levels in caregivers. Furthermore, interestingly, Rohde et al. [54] found that one-fourth of family members had evidence of anxious symptoms five years after the patient’s stroke incidents, and one-fifth of them exhibited anxiety symptoms. However, it must be highlighted that these symptoms were more related to the patient’s cognitive decline and not to personal burden levels. Finally, Mei et al. [57] found a positive association between caregivers’ moderate burden levels and the development of depression and anxiety symptoms.

Furthermore, studies have shown that informal caregivers who experience significant amounts of burden during their caregiving duties are at high risk of mental disorders as they score low on scales relating to their mental state and well-being [25,46]. Additionally, Kalavina et al. [45] recognized that most of the caregivers were distressed due to the lack of time and the patient’s entire dependence on the caregiver.

Moreover, two more studies focused their attention on caregiver burden during the time of COVID-19. Results showed that the pandemic intensified the caregiver’s distress, increasing the chances of developing and experiencing depressive and anxiety symptoms [36,39]. Finally, a study by Malhotra et al. [55], highlighted that younger caregivers had low positive feelings in terms of their capabilities and the outcome of their caregiving efforts in comparison to older caregivers who were more optimistic.

However, some studies have resulted in interesting findings. For instance, it was found that caregivers’ high level of benefit finding could predict low levels of anxiety, depression, and burden [57]. Additionally, a study by Koh et al. [48] found that levels of depression decreased statistically significantly over a 9-month period. As previously mentioned for this study, caregivers’ depressive symptom assessments were conducted at 3 and 12 months after the patient’s stroke. This reduction was attributed to caregivers becoming more familiar with their role and receiving more support from their relatives [58]. Furthermore, they noticed that if the caregiver, despite their burden, had a good relationship with the patient and had extra support, their depressive symptomatology was low [48]. Finally, in a qualitative study, all participants, despite their burden level, reported benefits from caring for the stroke survivor. For example, caregivers mentioned that they found ways to cope with stress, felt useful, accepted reality, learned to cherish everything, appreciated life, and cultivated a careful and cautious character [53].

### 4.5. Mechanisms Addressing Caregivers’ Burden

In the present review, some mechanisms are highlighted in different countries to address caregiver burden. For instance, in the discussion of financial issues among stroke caregivers, studies such as those by Hu [27] and Farahani [40] shed light on the economic strain experienced by caregivers due to reduced employment opportunities and high healthcare expenses. Contrastingly, caregivers from families with medical insurance payments, as mentioned in Hu’s study, tend to experience less anxiety and depression. This indicates that countries with comprehensive medical insurance coverage or robust healthcare systems may alleviate some financial burdens on caregivers. Moreover, the discussion on the impact of caregiver burden on psychological health, as evidenced by studies like those by Koh et al. [48] and Mei et al. [57], underscores the importance of support mechanisms. Koh et al. [48] found that depressive symptomatology decreased over time, attributing it to caregivers becoming more familiar with their role and receiving support from relatives. This suggests that countries with strong familial support networks or caregiver education programs may facilitate better psychological outcomes for caregivers. Furthermore, the cultural perspective highlighted in studies such as Wagachchige Muthucumarana’s [29] exploration of the Sri Lankan context underscores how cultural norms influence caregiver experiences. Understanding these cultural nuances can inform the development of culturally sensitive support mechanisms tailored to specific cultural contexts.

### 4.6. The Concept of the Caregiver

The concept of caregivers and the variables surrounding it encompass a broad array of factors that influence their experiences and well-being. The variables that surround the concept of caregivers include demographic characteristics such as age, gender, relationship to the care recipient, and socioeconomic status. For example, studies like those by Marima [46] highlight how caregivers in low-resourced settings face unique challenges due to financial constraints and lack of access to support services. Additionally, the study by Akosile [31] underscores how cultural norms and familial expectations shape the caregiving experience, with differing support dynamics between family members and friends.

Moreover, the discussion emphasizes the multifaceted nature of caregiver burden, encompassing physical health issues such as chronic fatigue, pain, and sleep disturbances, as evidenced by studies like those by Long [25], Tsai [30], and Marima [46]. Caregivers’ social functioning is also affected, with studies indicating reduced engagement in personal, work, and social activities due to caregiving demands [19,27,28,29,31,37,40,53]. Financial challenges, including reduced employment opportunities and high medical expenses, further compound the burden on caregivers, as highlighted in various studies [27,29,30,35,40,46].

Psychological aspects of caregiving are also prominent, with studies demonstrating a positive correlation between caregiver burden and the development of depressive and anxiety symptoms [34,41,48,51,54,57]. The impact of caregiving on mental health is significant, particularly among caregivers who lack sufficient support and resources to cope with the demands of caregiving [25,46,55].

Additionally, examining the nature of the caregiver–patient relationship, including communication patterns, mutual support mechanisms, and the level of dependency of the stroke survivor on the caregiver, offers crucial insights into how these relational dynamics influence caregiver well-being and the overall caregiving experience. By addressing these theoretical aspects comprehensively, we can develop more targeted interventions and support programs that are better aligned with caregivers’ specific needs and circumstances, ultimately enhancing their well-being and the quality of care provided to stroke survivors.

### 4.7. Strengths and Limitations

The review provides a comprehensive overview of the literature on caregiver burden in stroke patients, covering various aspects such as physical health, social functioning, financial issues, and psychological health. This breadth of coverage allows for a holistic understanding of the challenges faced by caregivers. Moreover, it focuses on studies published within the last five years, ensuring that the information is up-to-date and reflects the current state of research in this field. This study includes a diverse range of study types, including quantitative, qualitative, and mixed-methods research, providing a well-rounded perspective on caregiver burden. The inclusion of studies from different countries adds a global dimension to the review, highlighting how caregiver burden can vary across cultures and healthcare systems. Finally, it provides detailed analyses of the impact of caregiver burden on various aspects of caregivers’ lives. This in-depth analysis allows for a nuanced understanding of the topic. In this review, the quality assessment was a crucial step in evaluating the methodological rigor and potential bias across the included studies. The Newcastle–Ottawa scale (NOS) was employed to systematically assess the quality of non-randomized studies. By applying this scale, the review authors systematically evaluated each study’s quality based on criteria related to selection, comparability, and outcome assessment. The allocation of stars to each study allowed for a quantitative assessment of their methodological quality. This comprehensive quality assessment enabled the reviewers to categorize studies as having high, moderate, or low quality based on their total score, providing a clear indication of the overall strength of the evidence. The quality assessment was instrumental in ensuring that only studies with a sufficient level of methodological rigor were included in the review, thereby enhancing the reliability of the conclusions drawn from the synthesized evidence. It also facilitated a nuanced interpretation of the findings by considering potential biases and limitations in individual studies while determining their overall contribution to the review’s overarching conclusions. Hence some limitations should be stated. No statistical analysis or meta-analysis was performed due to the high level of heterogeneity among the studies. The review primarily focuses on describing the burden experienced by caregivers but does not delve into potential interventions or strategies to mitigate this burden.

The predominance of studies conducted in China (7/34 studies) raises important considerations regarding how cultural and lifestyle factors may influence the authors’ conclusions on caregiver burden in stroke care. Chinese culture places significant emphasis on familial duty and filial piety, where caregiving for family members, including stroke survivors, is often considered a moral obligation. This cultural context may influence caregivers’ perceptions of their roles, impacting their willingness to seek support or acknowledge the burden they experience. Additionally, the collectivist nature of Chinese society may shape caregiving dynamics, with extended family members often involved in caregiving responsibilities, potentially impacting the distribution of burden and available support networks. Moreover, lifestyle factors such as dietary habits, traditional medicine practices, and socioeconomic conditions may contribute to variations in caregivers’ experiences and the manifestation of burden. While the findings from Chinese studies provide valuable insights into caregiver burden within this cultural context, it is essential to acknowledge the potential limitations in generalizing these conclusions to diverse cultural settings. Future research should strive to incorporate a more diverse range of cultural perspectives to ensure a comprehensive understanding of caregiver burden and its implications globally.

Finally, it only includes articles published in the English language, which may exclude valuable research conducted in other languages, potentially limiting the comprehensiveness of the review.

## 5. Conclusions

**Figure 3:**
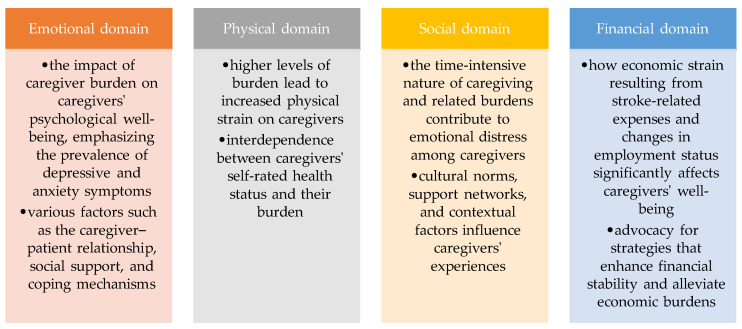
Main objective information per domain.

The conclusions drawn from this comprehensive review shed light on the multifaceted challenges faced by caregivers of stroke survivors. The review highlights that caregiver burden in the context of stroke care significantly impacts caregivers’ physical health, social functioning, financial well-being, and psychological health (Figure 3). Caregivers often experience a decline in their overall physical health, with fatigue, sleep disturbances, and chronic pain being common manifestations. Furthermore, the review underscores the emotional toll on caregivers, with anxiety and depression being prevalent outcomes, especially in cases of high burden. Financial constraints resulting from medical expenses and changes in employment status exacerbate the overall burden. The findings also emphasize the interplay between caregivers’ self-rated health and their burden, with better self-rated health mitigating the burden and vice versa. The review showcases how contextual factors, such as cultural norms and resource availability, can influence the caregiver experience. These conclusions are applicable not only in the healthcare sector but also in policy development and support systems for caregivers. Healthcare professionals can use these insights to provide tailored care and support to caregivers, recognizing that addressing caregiver burden is integral to stroke patient care. Therapists and healthcare professionals can use this knowledge to develop targeted interventions aimed at addressing the specific challenges identified in the review. For instance, physical therapists can incorporate strategies to alleviate caregiver fatigue and pain through tailored exercise programs and ergonomic education. Mental health professionals can focus on providing emotional support, stress management techniques, and interventions for depression and anxiety. Financial counselors can offer guidance on managing the economic burden and accessing available resources. Moreover, therapists can collaborate with caregivers to enhance their coping mechanisms, improve self-rated health, and strengthen their social support networks. By recognizing and addressing caregiver burden comprehensively within therapeutic settings, healthcare professionals can contribute significantly to the well-being of both caregivers and stroke survivors, ultimately fostering a more holistic and effective approach to stroke care and rehabilitation. Policymakers can consider these findings when developing programs and services to alleviate caregiver burden, ultimately improving the overall well-being of caregivers and enhancing the quality of care for stroke survivors.

### Future Pespectives: Enhancing Caregiver Well-Being

Looking ahead, there are promising avenues for improving the lives of caregivers and enhancing the overall quality of care for stroke survivors. Targeted interventions and support programs tailored to address the multifaceted challenges faced by caregivers, including physical health support, mental health assistance, and financial guidance, are essential. Integrating technology into caregiver support systems through telehealth services and mobile applications can increase accessibility and effectiveness. Community engagement and education initiatives, such as workshops and public awareness campaigns, can raise awareness and foster understanding and support within communities. Additionally, advocating for policy changes and increased funding for caregiver support programs and research can lead to tangible improvements in caregivers’ well-being. By incorporating these future perspectives into caregiver support systems and policy frameworks, we can make significant strides towards alleviating caregiver burden and promoting the overall well-being of both caregivers and stroke survivors.

## Figures and Tables

**Table 1:** Employed databases and search strategies.

Database	Search Strategy
PubMed	(“stroke” AND “caregivers” AND “burden”)
Scopus	(“stroke” AND “caregivers” AND “burden”)

**Table 2:** Quality assessment.

	Selection				Comparability		Outcome		
Study	Exposed Cohort	Non-Exposed Cohort	Ascertainment of Exposure	Outcome of Interest	Study Controls (a)	Study Controls (b)	Assessment of Outcome	Length of Follow-Up	Adequacy of Follow-Up
Okoye et al., 2019 [24]	√	-	√	√	√	√	√	-	-
Long et al., 2019 [25]	√	-	√	√	√	√	√	-	-
Zhu et al., 2019 [26]	√	-	√	√	√	√	√	√	√
Hu et al., 2018 [27]	√	-	√	√	√	√	√	-	-
Pucciarelli et al., 2019 [28]	√	-	√	√	√	√	√	√	√
Wagachchige Muthucumarana et al., 2018 [29]	-	-	-	√	√	√	√	-	-
Tsai et al., 2018 [30]	√	-	√	√	√	√	√	-	-
Akosile et al., 2018 [31]	√	-	√	√	√	√	√	-	-
Caro et al., 2018 [19]	√	-	√	√	√	√	√	-	-
Torregosa et al., 2018 [32]	-	-	-	√	√	-	√	-	-
Mei et al., 2018 [33]	√	-	√	√	√	√	√	-	-
Dou et al., 2018 [34]	√	-	√	√	√	√	√	-	-
Cao et al., 2022 [35]	√	-	√	√	√	√	√	-	-
Liu et al., 2022 [36]	√	-	√	√	√	√	√	-	-
Kavga et al., 2021 [37]	√	-	√	√	√	√	√	-	-
Kazemi et al., 2021 [38]	√	-	√	√	√	√	√	-	-
Lee et al., 2021 [39]	√	-	-	√	√	√	√	-	-
Farahani et al., 2020 [40]	√	-	√	√	√	√	√	√	√
Achilike et al., 2020 [41]	√	-	√	√	√	√	√	-	-
Barral et al., 2021 [42]	√	-	√	√	√	-	√	-	-
Formica et al., 2020 [43]	√	-	√	√	√	-	√	-	-
McCarthy et al., 2020 [44]	√	-	-	√	√	√	√	-	-
Kalavina et al., 2019 [45]	√	-	-	√	√	√	√	-	-
Marima et al., 2019 [46]	√	-	√	√	√	√	√	-	-
Wu et al., 2019 [47]	√	√	√	√	√	√	√	-	-
Koh et al., 2022 [48]	√	√	√	√	√	√	√	√	√
Kitoko et al., 2022 [49]	√	√	√	√	√	√	√	-	-
Wang et al., 2021 [50]	√	-	√	√	√	√	√	√	√
Chung et al., 2021 [51]	√	√	√	√	√	√	√	√	√
Freytes et al., 2021 [52]	√	-	√	√	√	√	√	-	-
Mei et al., 2020 [53]	√	-	-	√	√	√	-	-	-
Rohde et al., 2019 [54]	√	√	-	√	√	√	√	-	-
Malhotra et al., 2018 [55]	√	-	√	√	√	√	√	√	√
Oliva-Moreno et al., 2018 [56]	√	-	√	√	√	√	√	√	√

**Table 3:** Summary of included studies. M: Male; F: Female; N/A: not available.

First Author (Year)	Title	Type of Research (Quantitative—Qualitative)	N (Caregivers)	Time after Stroke	Type of Caregiver (Formal—Informal)	Caregiver Characteristics (Stay at Home or Not, Hours Spent with SS, etc.)	Measurement Scales for Burden
Okoye et al., 2019 [24]	Informal caregivers’ well-being and care recipients’ quality of life and community reintegration—findings from a stroke survivor sample	Quantitative	82 (31 M/51 F) Mean age = 36.13 ± 13.69	At least 1 month post-discharge 1–102 months average months = 15.32 ± 15.96	Informal 28—spouse 37—child 13—siblings and others 4—paid	- More female caregivers (62.2%) - 97.5% of the caregivers had at least a secondary-level education - 79.20% of the caregivers were the children or spouses of the survivors - The caregivers spent an average time of 16.5 ± 3.50 h per day in their role	Caregivers Strain Index (CSI)
Long et al., 2019 [25]	Factors predicting the health status of caregivers of stroke survivors: A cross-sectional study	Mixed methods	126 (36 M/90 F) Mean age = 52.4 ± 8.8	At least 1 month post-stroke	Informal 71—spouse 44—child 11—other (parent, grandchild, sibling)	- More female caregivers (71.4%) - 66.7% of the caregivers had general education level (elementary, secondary, or high-school - Half of the caregivers interviewed, caregiving duration ranged from 3 weeks to 6 months - 31.7% of the caregivers spent 6–12 months - 18.3% of the caregivers spent >12 months	- Modified Barthel Index - Zarit Burden Interview Scale - Multidimensional Scale of Perceived Social Support - Family Caregiver Conflict Scale - Short Form-36 Health Survey
Zhu et al., 2019 [26]	Determinants of caregiver burden of patients with haemorrhagic stroke in China	Mixed methods	202 (114 M/88 F) - Male mean age = 49.6 ± 1.2 - Female mean age = 48.2 ± 1.3	T1–T2–T3 T1: 1–2 before discharge T2: 3 months post-discharge T3: 6 months post-discharge	Informal	Living together: - T1 = 172 Y/30 N - T2 = 168 Y/34 N - T3 = 170 Y/32 N	Bakas Caregiving Outcome Scale (BCOS)
Hu et al., 2018 [27]	Relationship between the anxiety/depression and care burden of the major caregiver of stroke patients	Quantitative	117 (45 M/72 F) Mean age = 56.66 ± 9.62	N/A	Informal 76—spouse 35—child 6—other relatives	Care time: <4 h/d: 23 people 5–8 h/d: 37 people 9–12 h/d: 32 people >13 h/d: 25 people	- Hamilton anxiety scale - Hamilton depression scale - Zarit caregiver burden interview
Pucciarelli et al., 2019 [28]	Quality of Life Trajectories Among Stroke Survivors and the Related Changes in Caregiver Outcomes: A Growth Mixture Study	Quantitative	244 (34.8% M/65.2% F) Mean age = 52.7	T0: predischarge T1: 3 months post-stroke T2: 6 months post-stroke T3: 9 months post-stroke T4: 12 months post-stroke	Informal	- 65.2% of caregivers were females, with a medium to low educational level (44.2%) - The caregivers provided an average of 32.1 ± 32.8 h of informal care per week	- Caregiver Burden Inventory (CBI) - Hospital Anxiety and Depression Scale (HADS)
Wagachchige Muthucumarana et al., 2018 [29]	Caring for stroke survivors: experiences of family caregivers in Sri Lanka—a qualitative study	Qualitative	10 (2 M/8 F) Age range = 33–69 Mean age = 51	Within a month	Informal 6—wife 2—son 1—daughter 1—daughter-in-law	Duration of experience as the family caregiver: - 1 year = 4 caregivers - 1.5 years = 3 caregivers - 2 years = 1 caregiver - 2.5 years = 1 caregiver 1 missing Financial assistance: Y = 0/N = 10	Interviews Open-ended questions
Tsai et al., 2018 [30]	Mediating effects of burden on quality of life for caregivers of first-time stroke patients discharged from the hospital within one year	Quantitative	126 Mean age = 49 ± 13.2 Range = 20–81	Within a year Mean time since stroke = 154.8 days ± 88.8 Range = 4–315 days	Informal 44.4%—child 42.1%—spouse	- 83.3% living with other family members - 67.5% of caregivers were females 103—DID NOT hire a non-family caregiver 23—hired a non-family caregiver Financial assistance—Payer for medical fees 46—the patients themselves 66—their children 10—their spouse 4—other	Caregiver Strain Index (CSI)
Akosile et al., 2018 [31]	Informal caregiving burden and perceived social support in an acute stroke care facility	Quantitative	56 (21 M/35 F) Mean age = 28.2 ± 9.5	In acute phase	Informal	N/A	- Caregiver Strain Index (CSI) - Multidimensional Scale of Perceived Social Support
Caro et al., 2018 [19]	Burden and quality of life of family caregivers of stroke patients	Quantitative	30 (3 M/27 F) Mean age = 58.70 ± 13.34	Within a year	Informal 17—wife 7—child 2—mother 4—other	Living together: Y = 26/N = 4	Zarit Burden Interview Scale (ZBIS)
Torregosa et al., 2018 [32]	Dealing with stroke: Perspectives from stroke survivors and stroke caregivers from an underserved Hispanic community	Qualitative	8 Mean age = 53.25	N/A	Informal 6—wife or child 2—mother or sister	Living together: Y = 8/N = 0	Semi-structured interviews
Mei et al., 2018 [33]	Benefit finding for Chinese family caregivers of community-dwelling stroke survivors: A cross-sectional study.	Quantitative	145 (43 M/102 F) Mean age = 50.38 ± 13.29	N/A	Informal 52—spouse 73—child 15—parent 5—other	Caregiving hours per day: <8: 26 9–12: 27 >12: 92	Chinese Caregiver Burden Inventory (CBI)
Dou et al., 2018 [34]	Post-stroke depression as a predictor of caregivers burden of acute ischemic stroke patients in China.	Quantitative	271 (109 M/162 F) Mean age = 48.4 ± 14.7	In hospital	Informal 115—spouse 131—child 25—other members	primary caregiver	Zarit Caregiver Burden Interview (ZCBI)
Cao et al., 2022 [35]	A survey of caregiver burden for stroke survivors in non-teaching hospitals in Western China	Quantitative	328 mean age = 64.9 (13.4)	In hospital	Informal	primary caregiver	Zarit Burden Interview Scale (ZBIS), Social support Rating Scale (SSRS), General Self Efficacy Scale (GSES)
Liu et al., 2022 [36]	Burdens on caregivers of patients with stroke during a pandemic: relationships with support satisfaction, psychological distress, and fear of COVID-19	Quantitative	171	In hospital	Informal	N/A	Zarit Burden Interview Scale, Depression, Anxiety, Stress Scale (DASS-21), satisfaction of support survey, and Fear of COVID-19 Scale.
Kavga et al., 2021 [37]	The Effects of Patients’ and Caregivers’ Characteristics on the Burden of Families Caring for Stroke Survivors	Quantitative	109 stroke patients mean age 69.3 (13.7), 109 primary caregivers mean age 58.0 (13.5)	4 months since the stroke occurred	informal	to have the main responsibility for patient care, and to be living with the patient.	Barthel Index, the caregiving outcome (revised Bakas Caregiving Outcomes Scale), the caregiver’s mental state (Center for Epidemiological Studies-Depression, CES-D), the level of social support (Personal Resource Questionnaire, PRQ 2000)
Kazemi et al., 2021 [38]	Caregiver burden and coping strategies in caregivers of older patients with stroke	Quantitative	110 mean age = 32.09 ± 8.70	N/A	Informal	principal caregiver for a minimum of 1 month, not being paid for the care provided, and having a family relationship with the older patient.	Zarit Burden Interview (ZBI), Lazarus coping strategies questionnaires, and demographic checklists
Lee et al., 2021 [39]	Qualitative Study of Chinese Stroke Caregivers’ Caregiving Experience During the COVID-19 Pandemic	Qualitative	25 mean age 55.96 (11.28)	N/A	Informal	- primary adult caregiver of a stroke survivor with any level of disability - provided care for >1 month during the COVID-19 pandemic - were not diagnosed with any psychiatric disorder - were recruited from local community care and support centers	- Individual semistructured interviews—Barthel
Farahani et al., 2020 [40]	Investigating the needs of family caregivers of older stroke patients: a longitudinal study in Iran	Quantitative	210 mean age 42.8 ± 11.79	In hospital	Informal	- must be present at the hospital as the older patient’s companion at the time of admission, - assume the main responsibility of providing care to the older stroke patient at home	- Needs questionnaire for family caregivers of patients with stroke—Barthel index
Achilike et al., 2020 [41]	Caregiver Burden and Associated Factors Among Informal Caregivers of Stroke Survivors	Quantitative	88 mean age 54.33 ±13.65	within 2 years	Informal	- unpaid	Zarit Burden Interview, Patient Health Questionnaire-9, Barthel Index.
Barral et al., 2021 [42]	Patients’ productivity losses and informal care costs related to ischemic stroke: a French population-based study	Qualitative	108 (40 M/68 F), mean age 59.5 ± 15.9	1 year post-stroke	Informal 66—spouse 31—children 2—sibling 2—in-laws 7—other	Staying together: 80—Y/28—N	Self-reported questionnaires—IC Telephone interview
Formica et al., 2020 [43]	Factors related to cognitive reserve among caregivers of severe acquired brain injury	Quantitative	29	In hospital	Informal	N/A	- Repeatable Battery for the Assessment of Neuropsychological Status (RBANS), - Perceived Stress Scale (PSS), - Caregiver Reaction Assessment (CRA), - Cognitive Reserve Index—questionnaire (CRIq), - General Self-Efficacy Scale (GS), - Intolerance of Uncertainty Scale-12 (IUS-12)
McCarthy et al., 2020 [44]	Interpersonal relationship challenges among stroke survivors and family caregivers	Qualitative	19 care dyads, mean age = 55.61	Within 12 months of stroke	Informal	having experience with stroke and being in a committed care partnership, as well as by dyad type, living together in the community or spending a “significant amount of time together in care-related activities”	semi-structured interview guide (The Systemic-Transactional Model of Stress and Coping and the Developmental-Contextual Model of Couples Coping with Chronic Illness)
Kalavina et al., 2019 [45]	The challenges and experiences of stroke patients and their spouses in Blantyre, Malawi	Qualitative	18 (nine dyads of patients with stroke and spouses)	Sub-acute, chronic	Informal	marriage (only married couples who were living together at the time the stroke occurred were recruited	Semi-structured in-depth interviews and focus group discussions
Marima et al., 2019 [46]	Correlates of social support on report of probable common mental disorders in Zimbabwean informal caregivers of patients with stroke: a cross-sectional survey	Quantitative	71 mean age = 41.5 (13.8)	Acute, sub-acute	Informal	primary, unpaid caregivers	- Shona Symptoms Questionnaire (SSQ) - Multidimensional Scale of Perceived Social Support (MSPSS)
Wu et al., 2019 [47]	Relationship Consensus and Caregiver Burden in Adults with Cognitive Impairments 6 Months Following Stroke	Quantitative	60 dyads caregiver age: 59.18 (10.45), care recipient age: 65.87 (13.23)	N/A	Informal [spouses (58.3%) or children (23.3%)]	lived with care recipients	- Zarit Burden Interview (ZBI) - Quick Executive Interview (Quick-EXIT) - Abbreviated Dyadic Adjustment Scale (DAS-7)
Koh et al., 2022 [48]	The associations between caregivers’ psychosocial characteristics and caregivers’ depressive symptoms in stroke settings: a cohort study	Quantitative	214	Acute phase	informal	caregiver that remained during the first year after stroke	- caregivers’ depressive symptoms, measured using the 11-item Center for Epidemiologic Studies Depression, Oberst Caregiving Burden Score (OCBS)
Kitoko et al., 2022 [49]	Psychological Burden in Stroke Survivors and Caregivers Dyads at the Rehabilitation Center of Kinshasa (Democratic Republic of Congo): A Cross-Sectional Study	quantitative	85 (27 M/58 F) Mean age = 42.3 ± 14.34	In rehabilitation centre	N/A	N/A	Zarit Burden Inventory, Hospital Anxiety and Depression Scale
Wang et al., 2021 [50]	Burden of informal care in stroke survivors and its determinants: a prospective observational study in an Asian setting	Quantitative	661	They used a dataset from a prospective cohort study	Informal	N/A	- Zarit Burden Interview, - Modified Rankin Scale, - NIHSS
Chung et al., 2021 [51]	Depressive Symptom Trajectories in Family Caregivers of stroke Survivors during first year of caregiving	quantitative	102 (35 M/67 F) Mean age = 58 ± 13.3	T0 = acute rehabilitation hospitalization T1 = 6–10 weeks post-discharge T2 = 1 year post-discharge T3 = 2 years post-discharge	Informal 94—spouse 8—other	They were living with the patient	- Center for epidemiologic studies-depression (CES-D) - Zarit Burden Interview (ZBI) - Family Assessment Device - Interpersonal Support Evaluation List (ISEL) - Medical Outcomes Study SF-36
Freytes et al., 2021 [52]	Types of stroke-related deficits and their impact on family caregiver’s depressive symptoms, burden and quality of life	Quantitative	*n* = 109	N/A	Informal	Females, living together with stroke survivors,	- GES-D, - Zarit, - Health-related Quality of Life (VR-12), “Problem Checklist” - Barthel Index
Mei et al., 2020 [53]	Benefits finding among Chinese family caregivers of stroke survivors: A qualitative descriptive study	Qualitative	20 (12 M/8 F) mean age = 58.6	N/A	Informal	Living together	Audio-recorded interviews lasted between 30 and 60 min, with an average of approximately 42 min. Six main themes and 14 subthemes
Rohde et al., 2019 [54]	Stroke survivor cognitive decline and psychological wellbeing of family caregivers five years post-stroke: a cross-sectional analysis	qualitative	78	(1) after 6 months (2) after 5 years	Informal	Family members only living with stroke survivors	- MoCA, - Informant Questionnaire on Cognitive Decline in the Elderly (IQCODE)
Malhotra et al., 2018 [55]	Trajectories of positive aspects of caregiving among family caregivers of stroke -survivors: the differential impact of stroke survivor disability	Quantitative	173 (38.2% M/61.8 F) mean age: 50 ± 12.2	6–8 months after stroke—3 Interviews	Informal	Family members	- a 9-item Positive Aspects, - Barthel Index of Caregiving Scale (PAC)
Oliva-Moreno et al., 2018 [56]	Determinants of informal care, burden and risk of burnout in caregivers of stroke survivors the CONOCES Study.	Quantitative	3 months post-stroke: 224 mean age 55.2 12 months post-stroke: 202 mean age, 56.3	3 and 12 months post-stroke	Informal	Living at home, Female 70.5%–>3 months,	- Barthel Index, - Zarit Test, - HRQoL

**Table 4:** Domains of burden expression. N/A: not available.

First Author (Year)	Title	Emotional Domain	Physical Domain	Social Domain	Financial Domain
Okoye et al., 2019 [24]	Informal caregivers’ well-being and care recipients’ quality of life and community reintegration—findings from a stroke survivor sample	- The informal caregivers were reported to have significant burden - Moderate to high QoL for most of the caregivers (95.1%)	N/A	N/A	N/A
Long et al., 2019 [25]	Factors predicting the health status of caregivers of stroke survivors: A cross-sectional study	In relation to mental health, the low scores were present in all sub-scales, including social functioning, vitality, role-emotional, and general mental health.	Caregiver burden was the strongest predictor, which explained 70.3% of variations in health status of caregivers of stroke survivors.	It is possible that the lower scores on the mental health dimension could be related to the high level of perceived caregiver burden, which involves the emotional, social, and well-being status of these caregivers.	N/A
Zhu et al., 2019 [26]	Determinants of caregiver burden of patients with haemorrhagic stroke in China	The caregiver burden decreased over time - Most caregivers experienced burden across the first 6 months after stroke - In Chinese populations, people rarely take the initiative to express emotions and seek psychological help. Finding value in caregiving work is a way to obtain self-approval and experience positive emotions to relieve the burden.	N/A	N/A	N/A
Hu et al., 2018 [27]	Relationship between the anxiety/depression and care burden of the major caregiver of stroke patients	- Caregivers had a high level of anxiety and depression. The level of anxiety and depression is associated with the severity of functional disability of the patients. - Gender, education, care time per day and medical payment method were related to the anxiety and depressive symptoms of caregivers.	The length of care time was positively correlated with the anxiety and depression scores.	N/A	The economic burden caused by the disease is an important influencing factor of the caregiver anxiety and depression.
Pucciarelli et al., 2019 [28]	Quality of Life Trajectories Among Stroke Survivors and the Related Changes in Caregiver Outcomes: A Growth Mixture Study	- The caregivers of stroke survivors in the high and slightly improving QoL trajectory had the lowest caregiver burden, as well as the lowest anxiety, and depression. The caregivers of survivors in the moderate and slightly worsening QoL trajectory had a consistently higher burden, anxiety, and depression. Finally, the caregivers of the survivors in the recovery trajectory exhibited significant improvements in their burden, anxiety, and depression over time.	N/A	N/A	N/A
Wagachchige Muthucumarana et al., 2018 [29]	Caring for stroke survivors: experiences of family caregivers in Sri Lanka—a qualitative study	Some of the family caregivers were looking after their small children together with the stroke survivor, which caused them to feel overburdened.	Some caregivers were experiencing physical problems such as back, leg, and neck pain, high BP, and tiredness. They were missing the freedom to rest or sleep sufficiently.	The caregivers’ workload was increased due to their role. Family caregivers felt more homebound, and missed attending even the most common social gatherings.	Many caregivers became dependent on other family members, relatives, neighbours, friends for support, finances or meals when the family income was affected.
Tsai et al., 2018 [30]	Mediating effects of burden on quality of life for caregivers of first-time stroke patients discharged from the hospital within one year	Caregiver burden and family resources were significant predictors of caregiver QoL. The higher the CSI score, the lower the caregiver’s QoL	The subscales in the physical and social domains had similar scores	N/A	The lowest CSI score was in the financial domain
Akosile et al., 2018 [31]	Informal caregiving burden and perceived social support in an acute stroke care facility	N/A	N/A	No significant association was found between the patient socio-demographics and burden. Participants’ score on the family domain of the social support scale was the only domain with a significant correlation with their scores on the burden scale.	N/A
Caro et al., 2018 [19]	Burden and quality of life of family caregivers of stroke patients	Moderate levels of burden were associated with increased scores for risk of depression and changes in emotional health. Other possible factors that trigger stress could be social isolation, relationship problems with SS, feelings of self-annulment.	A high prevalence of chronic conditions and psychosomatic problems were reported. Predominantly body aches, and psychiatric problems. The most frequent health problem reported was back pain, which may be associated with depression and sleep disorders.	The reduction in sleep and leisure time may have health consequences and result in social isolation, as well as reducing active involvement in other activities, with further detrimental health consequences and increased burden on the caregivers.	N/A
Torregosa et al., 2018 [32]	Dealing with stroke: Perspectives from stroke survivors and stroke caregivers from an underserved Hispanic community	Caregiving brought conflicting emotions to caregivers. Constant worry, hypervigilance, multitasking.	N/A	N/A	N/A
Mei et al., 2018 [33]	Benefit finding for Chinese family caregivers of community-dwelling stroke survivors: A cross-sectional study.	Benefit-finding mediated the relationships between caregiver burden, anxiety, and depression, but it did not represent a moderating role in the relationship between caregiver burden, anxiety, and depression.	N/A	N/A	N/A
Dou et al., 2018 [34]	Post-stroke depression as a predictor of caregivers’ burden of acute ischemic stroke patients in China.	Post- stroke depression was modestly associated with caregiver burden of acute ischemic patients.	Female gender and normal muscle strength were the predictive factors for caregiver burden.	N/A	N/A
Cao et al., 2022 [35]	A survey of caregiver burden for stroke survivors in non-teaching hospitals in Western China	The more severe the condition, the heavier the caregiver burden	N/A	Only 27.4% of caregivers received adequate social support, while only 20.7% of caregivers had high levels of self-efficacy.	The type of health insurance has no impact on reducing the burden of caregivers
Liu et al., 2022 [36]	Burdens on caregivers of patients with stroke during a pandemic: relationships with support satisfaction, psychological distress, and fear of COVID-19	The caregiver burden was negatively correlated with satisfaction with family support, but positively with psychological distress and the fear of COVID-19.	N/A	N/A	N/A
Kavga et al., 2021 [37]	The Effects of Patients’ and Caregivers’ Characteristics on the Burden of Families Caring for Stroke Survivors	The severity of the caregivers’ burden correlated positively with the severity of depression, and higher depressive symptoms were related to life changes for the worse	The caregivers’ perception of burden was lower for those caregivers in good health, but higher for those who provided many months and daily hours of care.	A significant correlation between social support and caregivers’ burden score was found	N/A
Kazemi et al., 2021 [38]	Caregiver burden and coping strategies in caregivers of older patients with stroke	The care burden reported by the majority of caregivers of stroke survivors was mild to moderate	N/A	N/A	
Lee et al., 2021 [39]	Qualitative Study of Chinese Stroke Caregivers’ Caregiving Experience During the COVID-19 Pandemic	Increased psychological and emotional burden was detected	Physical burden, increased risk and frequency of abuse were unveiled.	N/A	N/A
Farahani et al., 2020 [40]	Investigating the needs of family caregivers of older stroke patients: a longitudinal study in Iran	N/A	N/A	Needs under the dimensions of “professional support” and “health information” were identified	N/A
Achilike et al., 2020 [41]	Caregiver Burden and Associated Factors Among Informal Caregivers of Stroke Survivors	Depressive symptoms were highly correlated with caregiver burden	Physical health was associated with caregiver health issues, stroke type, anxiety, and depression, and mental health was associated with caregiver health issues	N/A	N/A
Barral et al., 2021 [42]	Patients’ productivity losses and informal care costs related to ischemic stroke: a French population-based study	N/A	N/A	N/A	IC and productivity losses of patients with IS during the first year represent a significant economic burden for society compared to direct costs.
Formica et al., 2020 [43]	Factors related to cognitive reserve among caregivers of severe acquired brain injury	- The caregiver burden and the level of general distress influenced the cognitive performance - If cognitive functions are impaired auto-efficacy decrease, causing a caregiver burden improvement	N/A	N/A	N/A
McCarthy et al., 2020 [44]	Interpersonal relationship challenges among stroke survivors and family caregivers	N/A	N/A	Social workers may have the opportunity to assist dyads with disrupting negative communication cycles, strengthening empathy and collaboration, and achieving a balance so that each person’s needs are met.	N/A
Kalavina et al., 2019 [45]	The challenges and experiences of stroke patients and their spouses in Blantyre, Malawi	Caregiving was most difficult when the patients had faced incontinence, speech impairment and problems with anger management	N/A	N/A	N/A
Marima et al., 2019 [46]	Correlates of social support on report of probable common mental disorders in Zimbabwean informal caregivers of patients with stroke: a cross-sectional survey	Informal caregivers of patients with stroke were at risk of common mental disorders	Symptoms of insomnia, feeling overwhelmed, thinking too deeply and feeling run down	Caregivers who received an adequate amount of social support were likely to exhibit better mental health	Only 18.3% of carers reported adequate finances, and having lower income was associated with greater risk of psychiatric morbidity
Wu et al., 2019 [47]	Relationship Consensus and Caregiver Burden in Adults with Cognitive Impairments 6 Months Following Stroke	N/A	N/A	An enhancement of relationship consensus and satisfaction may potentially reduce burden and risk of adverse health outcomes in caregivers.	N/A
Koh et al., 2022 [48]	The associations between caregivers’ psychosocial characteristics and caregivers’ depressive symptoms in stroke settings: a cohort study	The study identified subjective burden, quality of care relationship and expressive social support as significantly associated with caregivers’ depressive symptoms	N/A	N/A	N/A
Kitoko et al., 2022 [49]	Psychological Burden in Stroke Survivors and Caregivers Dyads at the Rehabilitation Center of Kinshasa (Democratic Republic of Congo): A Cross-Sectional Study	The majority of stroke survivors and caregivers dyads had a burden of depression ranging from mild to moderate.	N/A	N/A	N/A
Wang et al., 2021 [50]	Burden of informal care in stroke survivors and its determinants: a prospective observational study in an Asian setting	Informal care burden remains high up to 12 months post-stroke. Factors such as functional dependency, stroke severity, informal caregiver gender and co-caring with foreign domestic workers were associated with informal care burden.			
Chung et al., 2021 [51]	Depressive Symptom Trajectories in Family Caregivers of stroke Survivors during first year of caregiving	- The prevalence of depressive symptoms was substantial for caregivers during the first year. - A notable finding is that 1 of every 10 caregivers reports newly developed depressive symptoms after 1 year of caregiving	Caregivers have worsened perceived health during the first year of caregiving. In addition, we identified that caregivers with persistent depressive symptoms were the most vulnerable caregivers at risk of having poor health status after the first year of caregiving. Caregivers with high caregiving requirements are less likely to have adequate rest, find time for exercise, and have enough rest when sick.	Caregivers with persistent depressive symptoms were characterized by having the lowest levels of perceived availability of social support, and the unhealthiest level of general family function	N/A
Freytes et al., 2021 [52]	Types of stroke related deficits and their impact on family caregiver’s depressive symptoms, burden and quality of life	The Cognitive/Emotional deficits appear to impact caregiver well-being more than the Motor/Functional deficits.	The motor/functional deficits failed to significantly predict any of the caregiver outcomes	N/A	N/A
Mei et al., 2020 [53]	Benefits finding among Chinese family caregivers of stroke survivors: A qualitative descriptive study	Internal benefits: increases in knowledge and skills, the development of positive attitudes, and the development of a sense of worthiness and achievement	N/A	External benefits: family growth and gains in social support	N/A
Rohde et al., 2019 [54]	Stroke survivor cognitive decline and psychological wellbeing of family caregivers five years post -stroke: a cross-sectional analysis	Significantly increased levels of family member anxious and depressive symptoms were associated with stroke survivor cognitive decline	N/A	N/A	N/A
Malhotra et al., 2018 [55]	Trajectories of positive aspects of caregiving among family caregivers of stroke -survivors: the differential impact of stroke survivor disability	Increase in stroke-survivor disability was associated with a significant downward shift (reduction in positive aspects of caregiving) of the Persistently Low trajectory and a significant upward shift (increase in positive aspects of caregiving) of the Persistently High trajectory.	N/A	N/A	N/A
Oliva-Moreno et al., 2018 [56]	Determinants of informal care, burden and risk of burnout in caregivers of stroke survivors the CONOCES Study.	- When providing informal care, the burden borne by informal caregivers was associated with the number of caregiving hours provided. - Having moderate or severe stroke at discharge also increased the caregiver’s likelihood of being at a high risk of burnout, especially at 3 months poststroke.	N/A	N/A	N/A

## Data Availability

Data are contained within the article.
